# Can mathematical modelling solve the current Covid-19 crisis?

**DOI:** 10.1186/s12889-020-08671-z

**Published:** 2020-04-24

**Authors:** Jasmina Panovska-Griffiths

**Affiliations:** 1 Department of Applied Health Research, Institute of Epidemiology and Healthcare, UCL, London, UK; 2 Institute for Global Health, Institute of Epidemiology and Healthcare, University College London, London, UK; 3 The Queen’s College, Oxford University, Oxford, UK

## Abstract

Since COVID-19 transmission started in late January, mathematical modelling has been at the forefront of shaping the decisions around different non-pharmaceutical interventions to confine its’ spread in the UK and worldwide. This Editorial discusses the importance of modelling in understanding Covid-19 spread, highlights different modelling approaches and suggests that while modelling is important, no one model can give all the answers.

## Background

Mathematical modelling can be used to understanding how a virus spreads within a population. The essence of mathematical modelling lies in writing down a set of mathematical equations that mimic reality. These are then solved for certain values of the parameters within the equations. The solutions of the mathematical model can be refined when we use information that we already know about the virus spread, for example, available data on reported number of infections, the reported number of hospitalisations or the confirmed number of deaths due to the infection. This process of model refinement (or calibration) can be done a number of times until the solutions of the mathematical equations agree with what we already know about the virus spread. The calibrated model, can then be used to tell us more about future behavior of the virus spread. One outcome of mathematical models is the predicted epidemic curve representing the number of infections caused by the virus over time. Using different parameters in the model, which may illustrate different interventions, or calibrating the model against different data, can change the predicted epidemic curve.

## Main text

Since COVID-19 transmission started in late January, mathematical modelling has been at the forefront of shaping the decisions around different non-pharmaceutical interventions to confine its’ spread in the UK. One model in particular, developed by Neil Ferguson’s group at Imperial College London [1] has been widely quoted as the driving force behind the social-distancing measures implemented in the UK and worldwide in order to halt COVID-19 spread. As a mathematical modeller with vast experience in developing, parametrising, calibrating and using models to answer different policy questions, I have been excited with the power that this mathematical model has had. But at the same time, knowing that mathematical modeling is designed to simplify reality and answer specific questions using relevant subsets of data, I had wondered how robust this mathematical model is, especially when the dataset they have used is only days, possibly a couple of months, long. “A mathematical model is as good as the data it uses” is a common sentence used among mathematical modellers. This had definitely come to mind a number of times with the Imperial model suggesting that “ … optimal mitigation policies (combining home isolation of suspect cases, home quarantine of those living in the same household as suspect cases, and social distancing of the elderly and others at most risk of severe disease) might reduce peak healthcare demand by 2/3 and deaths by half. However, the resulting mitigated epidemic would still likely result in hundreds of thousands of deaths … ” And especially when the model predictions that 500,000 people may die from severe COVID-19 infections using a value of *R*_0_= 2.4 in the model with no interventions, had to be drastically revised to a possible 20,000 people dying from severe infection, and an increased *R*_0_ to be closer to 3 reported recently [2, 3].

Then a few days later, another mathematical model, developed by Sunetra Gupta’s group at Oxford University, was published on the pre-print server medRxiv [3], and seem to suggest that ongoing epidemics in the UK … started at least a month before the first reported death.

These seemingly differing opinions from two leading modelling groups, started a discussion on which model is more accurate in predicting COVID-19 spread. People started to wonder whether the seemingly different conclusions drawn exposed problems with using models for infectious diseases transmission as key drivers of policy decision making [4].

To move forward, this Editorial highlights that the key question is not “which model is correct” but that “both models are correct for answering subquestions” that together will build the big picture. It is therefore important to put these two models, and their conclusions, in the context of the big picture around COVID-19 spread and interventions to halt it.

The key point to note is that these two mathematical models perturbing the media are very different models. Ferguson et al. model [1] is a stochastic individual based model (IBM) that considers the infectiousness of each individual within the population as a function of the number of contacts within the household, work/study place and random contacts. In contrast, Gupta et al. model [5] is a classic deterministic “susceptible-infected-recovered (SIR)” model that averages the infectiousness across the population. Both types of models have been used historically across different infectious diseases [6] and both have advantages and disadvantages, with the modelling approach chosen often based on the preference of the modeller. Under exactly the same conditions, i.e. same datasets, same parameters, using same numerical software for simulations, they ought to converge to one another. They may not, as is the case for the Imperial and Oxford models, when they use different data.

The Imperial model is calibrated against a number of cumulative deaths in the UK or the US by 14th March 2020 [1], while the Oxford model is calibrated to the number of deaths in the first 15 days of non-zero deaths in Italy and the UK [5]. In addition, the two critical parameters for COVID-19 spread that we have been hearing so much about: *R*_0_ and CFR are different in the two models. *R*_0_ is the basic reproduction number quantifying the number of secondary infections emerging from a primary infection and characterising the transmissibility of the virus [7]. CFR is the case fatality rate or ratio describing the death rate from COVID-19 due to infection and is a measure for the infection-severity or fatality of the virus [7]. The Imperial model has used a mean value of *R*_0_ =2.4 to derive their initial conclusions, but revised it to closer to 3 [2] in the last couple of days. Instead of CFR, they used infection fatality rate (IFR) that applies to a disease outbreak, and is closely related to CFR, but includes asymptomatic and undiagnosed cases. The mean IFR value used in the Imperial model is 0.9% with a range between 0.002% in 0–9 years old and 9.3% in over 80 years old. The Oxford model, on the other hand uses a mean *R*_0_ value of either 2.25 or 2.75 for different scenarios and a mean CFR value of 0.14%. Using different values for infectiousness (*R*_0_) and fatality (CFR) of COVID-19 will inevitably generate different results.

Therefore, comparing the two models would be analogous to comparing “apples” with “pears”, colloquially speaking. Hence I suggest, that instead of comparing the two models, we ought to view these as parts of a puzzle that needs to be built to give the full picture of how best to tackle COVID-19 spread. Taking a more interpretational view of the conclusions of the two models, we may say that the Imperial model suggests that suppression interventions are key to “flattening the epidemic curve”. The Oxford model, on the other hand, suggests that since a large proportion of the population may have already had it, it is important to undertake a large-scale antibody testing of the population as soon as possible. But aren’t both of these conclusions relevant? May it be that large-blanket anti-body testing within the current mitigating strategy is the way forward? This will be important as we start to question whether the reported symptomatic cases are forming the majority of COVID-19 cases or whether there are there additional under-reported asymptomatic cases. In previous pandemics such as SARS, the number of asymptomatic or mildly symptomatic people after infection was quite low, making it easier to contact trace the infected cases and isolate them. We don’t yet have enough information as to whether this is the case with COVID-19. So will the “flattened epidemic curve” rise again once we come out of the quarantine adopted worldwide? Let's build a model and answer this!

## Conclusions

Mathematical modelling is a powerful tool for understanding transmission of Covid-19 and exploring different scenarios. But, instead of focusing on which model is correct, we should accept that “one model can not answer it all” and that we need more models that answer complementary subquestions that can piece together the jigsaw and halt COVID- 19 spread.

## Data Availability

Not applicable.

## Abbreviations

IBM: Individual based model
SIR: susceptible-infected-recovered
*R*_0_: basic reproduction number
CFR: Case fatality rate
IFR: Infection fatality rate

## References

[1] Ferguson NM, Laydon D, Nedjati-Gilani D, et al. Impact of non-pharmaceutical interventions (NPIs) to reduce COVID- 19 mortality and healthcare demand. Preprint assessed 26^th^ March 2020 and available at https://www.imperial.ac.uk/media/imperial-college/medicine/sph/ide/gida-fellowships/Imperial-College-COVID19-NPI-modelling-16-03-2020.pdf. 10.1007/s11538-020-00726-x PMC7140590 32270376

[2] Epidemiologist Behind Highly-Cited Coronavirus Model Drastically Downgrades Projection, Daily Wire, 26^th^ March 2020. Accessed 26^th^ March 2020. https://www.dailywire.com/news/epidemiologist-behind-highly-cited-coronavirus-model-admits-he-was-wrong-drastically-revises-model?fbclid=IwAR3cws28hA9OC4Cdl55_ve9mq0zEpTbwrZ4WcG5kMboxkXNuthHU100N194.

[3] UK has enough intensive care units for coronavirus, expert predicts. New Scientist, 25^th^ March 2020. Accessed 26^th^ March 2020. https://www.newscientist.com/article/2238578-uk-has-enough-intensive-care-units-for-coronavirus-expert-predicts/.

[4] Coronavirus exposes the problems and pitfalls of modelling, The Guardian, 25^th^ March 2020. https://www.theguardian.com/science/2020/mar/25/coronavirus-exposes-the-problems-and-pitfalls-of-modelling.

[5] Lourenço J, Paton R, Ghafari M, et al. Gupta S. Fundamental principles of epidemic spread highlight the immediate need for large-scale serological surveys to assess the stage of the SARS-CoV-2 epidemic. Preprint assessed 26^th^ March 2020 and available at https://www.medrxiv.org/content/10.1101/2020.03.24.20042291v1.

[6] Chowell G, Sattenspiel L, Bansal S, Viboud C (2016). Mathematical models to characterize early epidemic growth: a review. Phys Life Rev.

[7] Panovska-Griffiths J (2019). Are we prepared for the next influenza pandemic? Lessons from modelling different preparedness policies against four pandemic scenarios. J Theor Biol.

